# The role of work-family conflict and job role identification in moderated mediating the relationship between perceived supervisor support and employee proactive behaviors

**DOI:** 10.3389/fpsyg.2022.857713

**Published:** 2022-10-11

**Authors:** Zhicheng Wang

**Affiliations:** School of Economics and Management, Hainan Normal University, Haikou, China

**Keywords:** perceived supervisor support, work-family conflict, proactive behaviors, job role identification, work-family balance

## Abstract

In recent years, the outbreak and persistence of COVID-19 has greatly changed the way people work, and encouraging employees to work online from home has become a new form of work for organizations responding to the epidemic. Based on the W-HR model, this paper explored supervisor support as a situational resource in the context of online office, aiming to verify the changes in work-family status caused by individuals facing the background of supervisor support, and then relate employees’ proactive behavior. Meanwhile, the predicted moderating effect of job role identification on supervisor support and work-family conflict was verified by considering the role identification generated in the positive interaction between employees and supervisors as individual resources. In this study, 555 employees from enterprises in the provinces of Jiangsu and Guangdong were selected as the research participants, and data were recovered at two time nodes. The results show that: (1) Perceived supervisor support significantly relates employee proactive behavior. (2) Work-family conflict mediates the relationship between perceived supervisor support and employee proactive behavior. (3) Job role identification has a significant moderating effect on the relationship between perceived supervisor support and work-family conflict, and high level of job role identification moderated the mediating effect of work-family conflict on perceived supervisor support and employee proactive behavior significantly. This paper expands the research perspective of employee proactive behavior. It can be used as a reference for enterprises to formulate strategies to improve employee proactive behavior in the process of digital reform.

## Introduction

While the outbreak and spread of COVID-19 have brought great challenges to world economic activities, it has also greatly promoted global digital transformation. Working mode is no longer limited to offline office, and online office has become a new choice for organizations in the context of the normalized epidemic (Wang and Wen, 2020). On the one hand, the popularization of digitalization provides a new mode of communication and collaboration within and between organizations, so that cooperation and exchange are not limited by space. On the other hand, with the popularity of online office, the boundary between the work and non-work areas of employees in an organization is becoming increasingly blurred (Derks et al., 2016). The promotion and popularization of digitalization bring great convenience to organizations and individuals. However, it also creates new challenges for organizations and employees. The proliferation of online office tools has blurred the line between employees’ work and home roles. If an employee does not receive strong support from the organization, it may lead to an inability to balance work and family. It is difficult for employees in this state to carry out proactive behavior. Work-family researchers encourage the understanding and exploration of ways to solve work-family imbalance among employees at the organizational and individual levels (Yucel and Minnotte, 2017; Rofcanin et al., 2018, 2020; Gu and Wang, 2019; Kalliath et al., 2020; Ben-Uri et al., 2021; Dishon-Berkovits, 2021).

Organizational culture and policies affect employees’ recognition of the organization. Organizational culture and policies are often communicated through employees’ direct leader, so employees often regard supervisors as the spokesperson of the organization (Li and Ling, 2008). The perceived supervisor support refers to the degree to which employees perceive that they are valued and supported by their superiors. Supervisor support is derived from the importance that organizations and supervisors attach to employees’ work contributions and the satisfaction of their emotional needs (Kottke and Sharafinski, 1988). The spread of online work has blurred the line between employees’ work and family roles, bringing new challenges for employees to balance work and family. In an organization, if the supervisor can provide sufficient work support to employees, sufficiently respect their opinions and attach importance to their contributions, it will provide them with effective work situation resource support, which will help meet their psychological needs and help them balance work and family properly with sufficient resources. For the purpose of giving back to the organization and the supervisor, employees in this state will spontaneously show the proactive employee behaviors expected by the organization and the leader (Liu, 2011; Hammer et al., 2016; Carlson et al., 2018; Du et al., 2018; Bakker et al., 2019; Arefin et al., 2020; Chan et al., 2021). Work-family conflict is a kind of interrole conflict, which refers to the role pressure of work and family. To some extent, the participation of one role will become difficult to achieve due to the requirements of the other role (Beutell, 1985). Work-family state has two sides. In the workplace, work-family state is usually manifested as a negative opposite relationship, namely work-family conflict (WFC). With the popularity of online office, employees blur the boundary between working time and family time, and the increase of working time encroaches on family time, leading to conflicts. Take China as an example, with the aggravation of the aging problem and the three-child policy has been promoted, employees have to take on heavier family responsibilities. Although the emergence of COVID-19 has brought convenience to employees’ online work, it has intensified the conflict between their work and family roles. On the one hand, employees have to take care of their family roles, such as taking care of the elderly and children, and on the other hand, they have to deal with the increasingly heated workplace competition environment, which makes the work-family conflict of employees increasingly prominent. Under the background of epidemic normalization, how to help employees coordinate work-family issues and obtain the desired employee behavior is a common concern of organizations.

However, work-family conflict has not received enough attention in the field of organizational management. Previous studies mostly chose work-family conflict as the influencing factor of employee behavior, and explored the negative impact of work-family conflict (Bai et al., 2016; Smoktunowicz et al., 2017; Arefin et al., 2020; Kramer et al., 2020; Cui and Li, 2021; Nazim et al., 2021; Park et al., 2021). Few studies have explored how to alleviate and resolve work-family conflict from the perspective of organizational support. Can employees alleviate work-family conflict with the support and attention of their supervisors? Can support and respect from supervisors motivate innovative behavior and organizational citizenship behavior? After the employee feels the support and respect from the supervisor, in the process of positive interaction between the two sides, can the employee obtain and stimulate the job role identification? Can employee’s job role identification be a boundary condition between supervisor support and employee work-family conflict? Under the background of building a harmonious work-family relationship, it is a valuable and urgent issue to be discussed.

Work-family relationships are complex and diverse. How can work conflict and help families? Why is family conflict at work, and why can it be beneficial? To answer the above questions, Brummelhuis and Bakker (2012) proposed a theoretical framework to explain positive and negative work-family processes based on resource conservation theory (Hobfoll, 1989). The Work Home Resources Model (W-HR Model) (Brummelhuis and Bakker, 2012). The W-HR model points out two types of resources: “individual resources” and “situational resources.” Among them, individual resources come from the inside of individuals, mainly including physical, psychological, intellectual, emotion and capital resources. Situational resources are outside the self and can be obtained from the individual’s social situation, including marriage, employment relationship, social network and other conditional factors, as well as social support such as respect, help and advice. In order to expand the existing research results, based on the W-HR model, from the perspective of situational resources and individual resources, this paper considers the supervisor support in an individual’s organizational relationship as a kind of situational resources, and explores the extent to which employees can alleviate work-family conflict by receiving the support and attention from the supervisor. To explore whether the acquisition of job role identification can encourage individuals to adopt more positive and effective coping styles to intervene in the relationship between supervisor support and employees’ work-family conflict, we consider the job role identification acquired during the interaction between employees and supervisors as individual resources. Therefore, this paper explains the logical relationship between “resource-state-behavior” based on the W-HR model. With the increase of the care and attention of the superior supervisor in the organization, the interaction between the employee and the supervisor is conducive to the awakening of the employee’s job role identification, so that the employee can get two-way energy supplement from the situation and individual resources. The influence of supervisor support on employee proactive behavior was investigated, and the roles of job role identity and work-family conflict were also investigated.

This study provides a more comprehensive perspective for understanding the mechanism of work-family conflict and the relationship between supervisor support and employee proactive behavior. At the same time, it also provides a useful reference for the organization to intervene employee proactive behavior from the perspective of work-family integrated management, in order to further integrate the relevant theories and achievements in the field of labor relations, human resource management system and organizational behavior, and provide a new theoretical perspective and practical guidance for promoting employee proactive behavior.

## Literature review

### Perceived supervisor support and employee proactive behavior

Supervisors are seen as agents of the organization, and their words and actions affect how the organization is perceived by employees. Perceived supervisor support is a typical work situation resource, which refers to employees’ perception of supervisor’s support, encouragement and care (Menguc et al., 2013). Effective supervisor support will make employees identify and receive organizational goals and culture, make employees have a positive attitude toward the organization, meet the needs of employees for the sense of belonging, make employees have the willingness to work for the organization, motivate employees to work hard, and make behaviors conducive to the organization (Strauss et al., 2008; Tement, 2014; Hammer et al., 2016; Han et al., 2020; Gopalan et al., 2021). Based on W-HR model, supervisor support, as a kind of situational resource, can effectively improve employees’ job satisfaction and emotional commitment, and then positively affect employees’ job involvement, knowledge sharing and other employee behaviors conducive to the organization (Schaufeli and Bakker, 2004; Kuvaas and Dysvik, 2010). Employee proactive behavior refers to employees’ efforts to improve the environment or themselves in order to pursue positive results for the organization and individuals (Frese and Fay, 2001). Proactive behavior is a kind of conscious, goal-oriented and motivated behavior. Employee proactive behavior means that employees are able to go beyond the formal tasks assigned to them and work hard to develop their goals and solve problems in order to bring positive results for themselves and the organization. Organizational environmental factors can significantly influence employee behavior choice. Supervisor is the most direct and controllable driving force for employee proactive behavior. As an employee’s supervisor, setting a compelling future direction and establishing a supportive background can effectively promote employees’ proactive engagement. Supportive leadership, such as helping employees to self-direct and self-manage, can improve proactive behavior (Braun and Nieberle, 2017; Rofcanin et al., 2018; Heras et al., 2020). In addition, the manager can present a clear vision of development to stimulate proactive behavior (Wei and Pan, 2012; Chen and Ellis, 2021). Research shows that supportive leadership such as authentic leadership can be a key factor in promoting employee proactive behavior (Hu et al., 2018). As a situational resource, supervisor support provides support for employees at the organizational level through the supervisor as an agent, so that employees can fully feel the respect and support from the organization and feel that the organization respects their contributions to them. This provides sufficient situational resources for employees to perform positive behaviors. After employees feel the support from their supervisors, they will make more efforts to improve themselves, overcome the difficulties brought by environmental factors, and try to solve problems, so as to repay the support provided by the organization and superior supervisors. Based on this, the following hypotheses are proposed in this study.

H1: Perceived supervisor support is positively related to employee proactive behavior.

### The mediating role of work-family conflict

The W-HR model indicates that work situation resources affect individual resource status. When resources are scarce, people struggle to acquire or maintain resources to deal effectively with the complexities of the environment (Hobfoll, 1989). When employees are in a state of sufficient resources and can get a good return on their input, employees tend to use redundant resources to get more resources. In contrast, when an employee is in a state of resource depletion, the employee takes action to prevent further loss of surplus resources (Hobfoll, 1989). Work-family conflict is a common work-family state of employees in the study of organizational behavior. When employees face work-family conflict, they will think that the balance between work and family is not balanced. If they cannot get the same resource support from the organization or family, they will feel the lack of resources. Employees in a resource deficit state will take action to prevent further loss of individual resources. Research shows that innovation, knowledge sharing and other behaviors are mostly initiative behaviors that are not explicitly stipulated by organizations (Parker and Collins, 2010). Without the support of organization and family resources, employees facing conflicts will think that they do not have enough resources to carry out their own changes and overcome environmental difficulties. In order to maintain existing individual resources or prevent further loss of their own resources, employees will reduce the occurrence of proactive behavior. Studies have shown that work-family conflict, as a blocking stressor, reduces employees’ innovative behavior (Innstrand et al., 2008).

In the context of COVID-19, online working from home has become a new form of organizational work. The promotion and popularity of online office brings convenience to organizations and employees, but also brings new problems. Online office forms blur the line between work and home. The dual role of organization staff and family members makes staff pressure multiply and individual resource loss is serious. Faced with the new problems arising from the popularization of online office, it is necessary to explore effective solutions from the organizational level. Supervisor support is nested in the organizational system as a work situation resource. As the agent of the organization, the supervisor becomes the link and communication medium between the organization and employees. Supervisor support means that supervisors fully respect employees’ work contributions and provide necessary work support to employees. Supervisor support is derived from the work situation resources at the organizational level. Employees perceive the care, support and encouragement from their supervisors, which effectively supplement their individual resources, help to alleviate the conflict between work and family, and provide a guarantee for the further occurrence of proactive behaviors. Therefore, this study suggests that work-family conflict may mediate the relationship between perceived supervisor support and employee proactive behavior. When employees perceive support, encouragement and respect from their supervisors, work-family conflict will be reduced and employee initiative will be increased. Based on this, the following hypotheses are proposed in this study.

H2: Work-family conflict mediate the relationship between perceived supervisor support and employee proactive behavior.

### The moderating role of job role identification

Organizational identification refers to whether an individual’s self-identity is consistent with the concept of organizational identification (Ashforth and Mael, 1989; Ashforth et al., 2008). Existing research shows that the strength of personal identity to the organization, the more likely it is advantageous to the company’s action, because with the increase of identity, group members will not only focus on the needs of self, but will be trying to keep the consistency of itself and the interests of the enterprise, the enterprise’s development goals, as the extension of its own interests. Individuals are less inclined to place their own interests above those of the organization, or even to distinguish between the two (Mael and Ashforth, 1995; Dukerich et al., 2002; Edwards and Peccei, 2010; Avanzi et al., 2015; Cepale et al., 2021). Employee’s job role identification is the cognition formed on the basis of their organization identification. Employees’ job role identification has an impact on their role behavior. From the perspective of interaction, job role identification comes from a certain social structure, and individuals acquire the meaning of job role identification in the process of interaction with others (Ding et al., 2017). Role is an expectation of the organization to the individual. Based on the specific situation, the individual has the perception and recognition of how to internalize this expectation (Schwartz et al., 2011). When an individual’s attitude and behavior in the process of playing a role are consistent with the role expectation, it is called job role identification (Farmer et al., 2003). The research points out that job role identification is gradually formed in role playing, including how to select, confirm and play individual roles, and identify oneself as the way of expression of this role. Therefore, job role identification is a process of continuous shaping and strengthening under the influence of the environment (Renee, 2009). According to social identity theory, job role identification is based on group identity. In an organization, employees’ job role identification is the identification of the group identity and the knowledge system, value and emotion it represents. Emphasize the relationship between individual employees and their organizations (Dishon-Berkovits, 2021). Therefore, employees are motivated to seek and maintain a positive organizational identity, and once an employee’s organizational identity is established, it will have an impact on their individual behavior (Du et al., 2020). The supervisor, acting as an agent of the organization, provides employees with situational resources by supporting, encouraging and respecting their contributions. In this process, a positive interaction is formed between employees and their supervisors. In this interaction, employees seek and establish their own organizational identity and positioning, complete their job role identification process, and generate new individual resources. This helps to replenish the personal resources lost by employees in dealing with work-family conflicts and provides emotional and cognitive support for the occurrence of proactive behaviors of employees in the future.

Specifically, job role identification has the possibility to reduce the influence level of supervisor support on work-family conflict. Based on the identification of organizational identity, employees with strong sense of job role identification will make more efforts to change their own situation, overcome the difficulties brought by environmental factors, and gain their individual resources, thus effectively alleviating the work-family conflict level. The resources provided by employees in the state of job role identification can better meet the resource needs of employees, increase the sense of resource acquisition of employees, and enable them to participate in proactive behavior such as innovation with more active use of excess resources. When job role identification plays an effective role, individual resources of employees are at a high level, which to some extent inhibits the negative impact of supervisor support on work-family conflict. When the level of job role identification is low, employees have less personal resources to deal with corporate affairs and family affairs, and the negative impact of supervisor support on work-family conflict is more prominent. Based on this, the following hypotheses are proposed in this study.

H3: The higher the level of job role identification, the weaker the negative impact of perceived supervisor support on work-family conflict.

Based on the above discussion, job role identification moderates the relationship between supervisor’s sense of support and work-family conflict, and improves employees’ individual resources in the process of dealing with work-family problems. Job role identification is based on the interaction process to generate organizational identity, and then make positive behavior beneficial to the organization. Specifically, when employees are in work-family conflict, the individual resources provided by job role identification can help employees relieve their negative emotions in the process of work and in the process of dealing with work-family relationship, and avoid employees refusing to engage in positive behaviors by relieving their emotions through other behaviors. In other words, job role identification not only moderates the relationship between perceived supervisor support and work-family conflict, but also further moderates the mediating role of work-family conflict between perceived supervisor support and employee proactive behavior. Based on this, the following hypotheses are proposed in this study.

H4: Job role identification moderates the mediating role of work-family conflict between perceived supervisor support and employee proactive behavior, that is, the higher the level of job role identification, the stronger the mediating role of work-family conflict.

**FIGURE 1:**
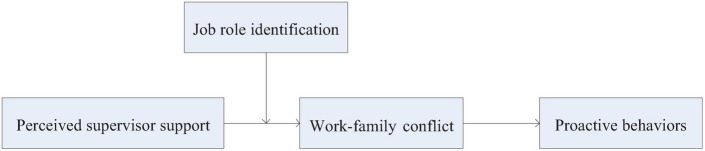
Theoretical model. SS, Perceived supervisor support; WFC, work-family conflict; JRI, job role identification.

The theoretical model constructed in this study is shown in Figure 1.

## Research methods

### Sample and collection

We collected our data from multiple organizations in the provinces of Jiangsu and Guangdong. We asked employees to report their perceived supervisor support, work-family conflict, job role identification and employee proactive behavior. We measured perceived supervisor support, work-family conflict and job role identification at time 1. Two weeks later, we measured employee proactive behavior. Data collection was performed in two ways: (1) By e-mail, mainly through the enterprise contact person who sent the link to the electronic version of the questionnaire and the answer instructions to the respondents; and (2) By on-the-spot recycling, in which members of the research group visited the enterprises, distributed questionnaires to the subjects, and collected them on the spot. A total of 650 questionnaires were collected in this survey. After eliminating the invalid questionnaires with missing answers and too many similar options, 555 valid questionnaires were obtained, with an effective recovery rate of 85.38%. The composition of the valid samples is shown in Table 1. It can be seen that the samples have a wide distribution and meet the basic requirements of the study. In terms of gender, 54.1% of the participants were male; 58.2% were aged 26–35; 58% were married; 55.7% held a bachelor’s degree or above; and 65.9% of participants had a monthly income of more than 4,000 Yuan.

**TABLE 1:** Composition of valid samples.

Name	Category	Number	Percent	Name	Category	Number	Percentage
Gender	Male	300	54.1	**Education**	High school or below	61	11
	Female	255	45.9		Vocational school/College	185	33.3
Age	25 years old	158	28.5		Undergraduate	277	49.9
	and below						
	26–35 years old	323	58.2		Master degree or above	32	5.8
	36–45 years old	65	11.7	Income	Below 2,000	0	0
	46 years old and above	9	1.6		2,001–4,000	189	34.1
Marriage	Unmarried	227	40.9		4,001–6,000	243	43.7
	Married	322	58		6,001–8,000	82	14.8
	Divorced	6	1.1		Over 8,000	41	7.4

### Measures

In this study, we selected appropriate scales available both at home and abroad, which have been widely used in China to ensure the reliability and effectiveness of the measurement variables. We translated these items from English into Chinese in accordance with the “translation and back translation” procedure. Except for the control variables, we used a 7-point Likert scale to measure all variables, ranging from 1 = not compliant to 7 = conforming.

#### Perceived supervisor support

We selected and revised the perceived supervisor support (SS) scale developed by Greenhaus et al. (1990) and formed a perceived supervisor support load scale with four items. A sample item is as follows: “Supervisors offer help when employees are in trouble.” The Cronbach’s α coefficient for perceived supervisor support was 0.822.

#### Work–family conflict

The scale used to measure work-family conflict (WFC) was adapted from Grzywacz and Marks (2000). The scale consists of eight items, four of which measure work-family conflict and four of which measure work-family facilitation. A sample item is as follows: “Stress at home makes you irritable at work.” The Cronbach’s α coefficient for work-family conflict was 0.933.

#### Job role identification

We selected and revised the job role identification (JRI) scale developed by Saleh and Hosek (1976) and formed a job role identification load scale with four items. A sample item is as follows: “I’m very committed to my role.” The Cronbach’s α coefficient for job role identification was 0.952.

#### Employee proactive behavior

We selected and revised the employee proactive behavior (PB) scale developed by Parker (Parker et al., 2006) and formed employee proactive behavior load scale with four items. A sample item is as follows: “I look for new ways to solve problems.” The Cronbach’s α coefficient for employee proactive behavior was 0.87.

Control variables. Considering that demographic variables may affect employees’ innovative behavior, we controlled gender, age, marriage, education and income (Tang et al., 2017). In terms of gender, 54.1% of the participants were male; 58.2% were aged 26–35; 58% were married; 55.7% held a bachelor’s degree or above; and 65.9% of participants had a monthly income of more than 4,000 Yuan. Refer to Table 1 for specific standards.

## Data analysis and results

### Common method bias test

Common method variance (CMV) refers to the artificial variation among variables caused by use of the same subjects or data sources, similar measurement situations, common project context, or project characteristics (Podsakoff et al., 2003). Although in this study we used a two time-point data collection method to control the common method variance problem, the fact that the items in each questionnaire were filled in by one person means that there could still be common method variance in the measurement process. In this study, Harman’s single factor test was used to test the degree of variation of the sample data. Four factors were extracted through principal component analysis. The results show that the variance explained by the first factor was 36.07%, less than the critical value of 40%. This indicates that the CMV of the data employed in this paper was not significant, although this issue deserves further investigation. Because the survey data of this study were filled in by the employees themselves, it was necessary to conduct a common method variance test. Using Harman’s single factor test method, all measurement items were included in a common factor for model fitting (see the single-factor model in Table 2). It can be seen from Table 2 that the single factor model fitting is poor, which indicates that the CMV of the questionnaire data in this study is relatively small.

**TABLE 2:** Confirmatory factor analysis.

Model	Model factor	χ^2^	*df*	χ^2^/*df*	NFI	RFI	IFI	TLI	CFI	RMSEA
Single factor	A+B+C+D	3,147.08	90	34.968	0.526	0.368	0.533	0.375	0.531	0.248
Two factors	A+B+C, D	2,500.156	89	28.092	0.624	0.492	0.632	0.501	0.63	0.221
Three factors	A+B, C, D	1,990.131	87	22.875	0.7	0.587	0.71	0.597	0.708	0.199
Four factors	A, B, C, D,	218.468	84	2.601	0.967	0.953	0.979	0.971	0.979	0.054

A = SS, Perceived supervisor support; B = WFC, work-family conflict; C = PB, proactive behaviors; D = JRI, job role identification.

### Confirmatory factor analysis

In this study, the software Amos 24 was used to conduct confirmatory factor analysis on four variables (i.e., perceived supervisor support, work-family conflict, employee proactive behavior and job role identification) to test the discriminant validity of the measurement variables (see Table 2). It can be seen from Table 2 that the four-factors model is the most suitable (χ2/df = 2.601; NFI = 0.967; TLI = 0.971; CFI = 0.979; RMSEA = 0.054), as it clearly performs better than the other models, indicating that the measurement variables in this study have good discriminant validity. One can see from Table 2 that the four-factors model has the best fit compared to other models, and each fitting index is at an acceptable level, indicating that the four main constructs in this study have good discriminative validity.

### Descriptive statistics

In this study, gender, age, marriage, education and income were included as control variables. Analysis showed that gender, marriage and education had no significant effect on the dependent variables. Age and income had significant effect on the dependent variables. The mean value, standard deviation, and correlation coefficient of each variable are shown in Table 3, with the square root of the average variance extracted (AVE) on the diagonal. According to Table 3, perceived supervisor support was positively correlated with employee proactive behavior (*r* = 0.566, *p* < 0.01). There was a significant negative correlation between perceived supervisor support and work-family conflict (*r* = −0.189, *p* < 0.01). Work-family conflict was negatively correlated with employee proactive behavior (*r* = −0.203, *p* < 0.01). This suggests that work-family conflict may have an incomplete mediating effect on the relationship between perceived supervisor support and employee proactive behavior. The results show that perceived supervisor support, work-family conflict, employee proactive behavior and job role identification were significantly correlated at a moderate level, which allowed us to further perform a regression model test. It can also be seen from Table 3 that the critical values of the correlation levels were not higher than 0.75. Therefore, there was no serious multicollinearity problem in the analysis of the data.

**TABLE 3:** Descriptive statistics and correlation coefficients of the variables.

	1	2	3	4	5	6	7	8	9
Gender	1								
Age	0.025	1							
Married	0.096*	0.605**	1						
Education	0.058	−0.053	−0.058	1					
Income	−0.084*	0.298**	0.208**	0.251**	1				
SS	−0.013	−0.032	0.016	0.026	0.080	1			
WFC	−0.073	−0.009	0.000	−0.055	−0.052	−0.189**	1		
PB	−0.010	0.090*	0.067	0.034	0.096*	0.566**	−0.203**	1	
JRI	0.024	0.049	0.081	0.053	0.129**	0.639**	−0.251**	0.582**	**1**
M	1.46	3.26	1.60	2.50	4.38	5.90	3.22	5.66	5.87
SD	0.50	1.20	0.51	0.77	1.64	1.07	1.68	0.97	1.15

*Indicates a significant correlation at 0.05 level (double tail); ** indicates a significant correlation at 0.01 level (double tail); *n* = 555.

SS, Perceived supervisor support; WFC, work-family conflict; PB, proactive behaviors; JRI, job role identification.

### Hypothesis tests

In this study, we used the process macro program of SPSS 23 and the bootstrap method to test the hypotheses (Preacher et al., 2007). This method is superior to more traditional methods because it does not require a normal sampling distribution but can instead use the ordinary least squares regression to estimate the direct and indirect effects of the mediations, and can use the 1,000 bias-correction guidance.

#### Main effect tests

The results of the main effect analysis of perceived supervisor support is shown in Table 4. According to Table 4, perceived supervisor support can significantly promote employee proactive behavior (β = 0.449, *p* < 0.001). Thus, H1 is supported.

**TABLE 4:** Standardized results of the main effects of the Perceived supervisor support.

Variable	Proactive behaviors	
	Coefficient	Standard error
Perceived supervisor support	0.499***	0.032

***P < 0.001.

#### Mediating effect of work-family conflict

First, the process macro program in SPSS 23.0 and the bootstrap method were used to test the mediating role of work-family conflict between perceived supervisor support and employee proactive behavior (see Table 5). It can be seen from Table 5 that the value of the mediating role of work-family conflict between perceived supervisor support and employee proactive behavior was 0.00633; moreover, the 95% confidence interval of bootstrap = 5,000 (0.0005, 0.00173) did not contain 0, thus indicating that the mediating role was significant. Thus, H2 is supported.

**TABLE 5:** Test results of the mediation effect of work-family conflict (*n* = 555).

Intermediate variable path	Mediating role value		Confidence interval (95%)	
	Coefficient	Standard error	BootLLCI	BootULCI
SS→WFC→PB	0.0063	0.0042	0.0005	0.0173

SS, Perceived supervisor support; WFC, work-family conflict; PB, proactive behaviors.

#### Moderating effect of job role identification

In this study, we used the macro program in SPSS 23.0 to test the moderating effect of job role identification on perceived supervisor support and work-family conflict (see Table 6). According to Table 6, job role identification had a significant moderating effect on the path from perceived supervisor support to work-family conflict (β = −0.1104 *p* < 0.05), indicating that job role identification has a negative regulatory effect between perceived supervisor support and work-family conflict. Thus, H3 is supported.

**TABLE 6:** Test results of the adjustment effect of job role identification (*n* = 555).

Adjustment term	Work-family conflict	
	Coefficient	Standard error
SS × JRI	−0.1104*	0.0432

SS, Perceived supervisor support; WFC, work-family conflict; JRI, job role identification. *Indicates a significant correlation at 0.05 level (double tail).

**FIGURE 2:**
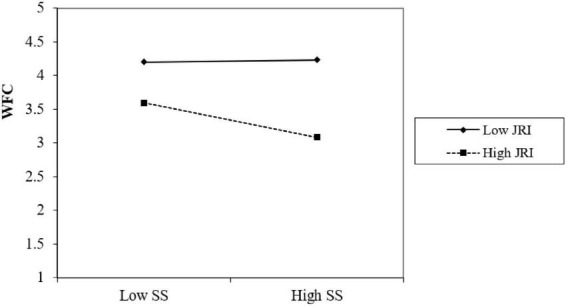
The moderating role of job role identification.

In order to understand the essence of the regulation effect between perceived supervisor support and work-family conflict more clearly, all the samples were divided into two groups depending on job role identification. In more detail, we considered the samples with low job role identification to be those with values of job role identification lower than the mean value minus the standard deviation, and the samples with high job role identification were defined as those having values of job role identification higher than the mean value plus the standard deviation. Then, the simple slope test and the simple effect analysis chart were drawn (see Figure 2).

It can be seen from Figure 2 that perceived supervisor support has a significant negative predictive effect on work-family conflict when job role identification is low, and a weakened predictive effect when job role identification is high. This shows that job role identification has a moderating role in this process, and hypothesis 3 is supported.

We further verified the mediating role of job role identification on work-family conflict between perceived supervisor support and employee proactive behavior (see Table 7). As can be seen from Table 7, when job role identification is one unit standard deviation higher, the 95% confidence interval of the difference of indirect effects did not include 0, indicating that the difference in the indirect effects was significant. Hypothesis 4 is partially supported. In other words, at a high level of job role identification, the mediating role of work-family conflict between supervisor support and employee proactive behavior is moderated.

**TABLE 7:** Bootstrap analysis of the moderated mediating role (5,000 samples).

Intermediate variable path	JRI	Effect value	BootSE	Confidence interval (95%)	
				BootLLCI	BootULCI
SS→WFC→PB	4.7316 (M-1 SD)	−0.0008	0.0067	−0.0179	0.0098
	5.8779 (M)	0.0064	0.0066	−0.0042	0.0233
	7 (M + 1 SD)	0.0135	0.0093	0.0001	0.0388

eff1/eff2/eff3 refer to a standard deviation below/equal to/higher than the mean value, respectively.

## Discussion

### Theoretical implications

The theoretical contributions of this study are as follows. First, we explored the relation of perceived supervisor support on employee proactive behavior and expanded the exploration of antecedent variables of employee proactive behavior. Previous studies mainly explored the antecedents of employee behavior, especially proactive behavior, from negative factors such as workplace ostracism (Zhao et al., 2013; Gürlek, 2021; Zahid et al., 2021; Zhou et al., 2021). In the context of COVID-19, the popularity and promotion of working from home has brought a series of problems to employees, such as conflicts between work and family roles. Based on the W-HR model, this study regarded supervisor support as a kind of work situation resource. As the agent of the organization, the supervisor’s support, encouragement and respect for the employees can make the employees feel the care from the organization, which provides enough resources for the employees to get rid of the affairs of the organization and deal with the relationship between work and family. Second, we extend the W-HR model. W-HR model focuses on the work-family domain of employees and provides a complete and focused theoretical framework for understanding the specific causes, linkage mechanisms and consequences of work-family interface (Chan et al., 2020). Based on the W-HR model, resources can be divided into individual resources and situational resources. When the demands of the work field consume personal resources and hinder individuals from contributing to the family field, work-family conflict will result. Individual resources come from within individuals, mainly including physical, mental, governance, emotional and capital resources. Situational resources can be obtained from individual situations such as marital relationships, employment relationships and social networks (Brummelhuis and Bakker, 2012). Previous studies have explored the impact of workplace resources on work-family status from the perspectives of organizational culture, psychological climate, leadership, and post characteristics (Wayne et al., 2004; Braun and Nieberle, 2017; Lee et al., 2017, 2019; Almeida et al., 2018; Brosch and Binnewies, 2018; Rofcanin et al., 2018; Heras et al., 2020; Postema et al., 2021). Previous studies have explored the influence of individual resources on work-family status from the perspectives of body, emotion, job involvement and psychological capital (Almeida et al., 2016, 2018; Carlson et al., 2018; Matei and Vrg, 2020; Postema et al., 2021). In this study, perceived supervisor support was used as work situation resource to enrich the content of work situation resource in W-HR model. As the agent of the organization, the supervisor can communicate and talk with the employees on behalf of the organization. The behaviors of the supervisor, such as encouraging, supporting and respecting the employees, can generate positive interaction between the supervisor and the employees. Such interaction can promote the employees to establish organizational identity and generate job role identification, thus forming new individual resources. As a boundary condition, employee’s job identification can continue to influence employee’s work and family status. The positive interaction between supervisors and employees makes the working situation resources and individual resources not isolated and forms a linkage effect. Our research enriched and expanded the W-HR model.

### Practical implications

Our findings have also some implications for management practice. The popularization and promotion of digitization urges enterprises to speed up the construction of a new human resource management model. The people-oriented concept is the key to construct a new human resource management model. Giving full play to the initiative of individual employees in order to achieve the sharing and iteration of knowledge, technology and other key information within the organization is the key factor for enterprises to continuously innovate and improve their competitiveness. Based on w-HR model, this study uses supervisor support and job role identification as work situation resources and individual resources, respectively, to verify the influencing factors of increasing employee proactive behavior. Our findings have implications for management practice.

First, from the organizational level. As the agent of the organization, the superior supervisor of the employee is the medium through which the organization communicates with the employee, exchanges and conveys organizational policies. In the context of the current epidemic, it is necessary to give full play to the role of supervisors, provide necessary support to employees, fully respect employees’ contributions to the organization, and encourage and motivate employees in the process of work. Supervisor support such as oral praise, flexible work arrangement, pro-family policy and friendly organizational atmosphere can make employees feel supported by the organization and free them from work affairs, so that they can deal with family problems more easily (Hammer et al., 2016; Lee et al., 2019; Peeters et al., 2020).

Second, from the perspective of employees. Employees can feel the support from the organization through the interaction and communication with their supervisors and the encouragement, support and respect from supervisors. This allows employees to identify with their organization and construct their own identity within the organization. Continued support from supervisor, can make the employee role identity, this aspect for employees to supplement, on its own resources and stop work family conflict, on the other hand, fully the role of identity will inspire employees to implement knowledge sharing, organizational citizenship behavior initiative and innovation of employee behavior, to these is beneficial to the organization’s behavior as a feedback of the organization’s support (Christ et al., 2011; Dechawatanapaisal, 2018).

### Limitations and future research

This study has some limitations. First, the survey data came directly from the employees. In view of this, in terms of employees’ behavior, follow-up research can measure the supervisors subjectively perceived employees’ proactive behavior and compare it with the employee’s perceived proactive behavior, to better understand the degree of employee’s innovative behavior more intuitively. In addition, although our research group repeatedly emphasized the confidentiality and the academic value of our questionnaire, there is still the possibility that employees were unwilling to report their actual situation regarding their work-family status. Therefore, follow-up research can reduce the research error as much as possible according to the actual situation and in the form of other reviews. In addition, the independent variables and dependent variable were collected, respectively, only at time 1 and time 2. This strongly limits inferences on the plausible causality relationships. Follow-up research can be conducted in different ways to achieve the purpose of reducing errors.

Second, this study focused only on the moderating role of the job role identification system, i.e., a situational factor, in the influence of the perceived supervisor support on work-family conflict. However, in fact, the job role identification system can also improve employees’ proactive behavior. In future research, we can further compare and explore the difference between job role identification and perceived supervisor support in the process of influencing employees’ proactive behavior.

Third, based on our general research question, the present study clarified the impact and mechanisms of the perceived supervisor support on employee proactive behavior; as such, the impact and process of the perceived supervisor support on employee proactive behavior remains to be clarified. Further in-depth and targeted research can be performed targeting different types of enterprises.

## Conclusion

The implementation of innovation-driven development strategies in China requires enterprises to construct new models of human resource management (HRM) to face increasing challenges and rapid changes in the digital era. Exploring the influencing factors of employee proactive behavior is the prerequisite to trigger employee innovative behavior. We get the following research conclusions. First, based on W-HR model, this study explored the relationship between perceived supervisor support, work-family conflict and employee proactive behavior. The moderating mechanism was tested in the context of job role identification. The main findings of this paper are as follows. First, perceived supervisor support can significantly increase employee proactive behavior. Second, work-family conflict mediates the relationship between supervisor support and employee proactive behavior. Thirdly, job role identification plays a moderating role between supervisor support and work-family conflict. At the same time, we verified that high level of employee’s job role identification moderated the mediating effect of work-family conflict on perceived supervisor support and employee proactive behavior, that is, the higher level of employee’s job role identification the stronger the mediating effect of work family conflict.

## Data availability statement

The original contributions presented in this study are included in the article/supplementary material, further inquiries can be directed to the corresponding author/s.

## Ethics statement

Written informed consent was obtained from the individual(s) for the publication of any potentially identifiable images or data included in this article.

## Author contributions

ZW contributed to conceptualization, formal analysis, investigation, methodology, supervision, writing—original draft preparation, funding acquisition, and read and agreed to the published version of the manuscript.
